# Effectiveness and tolerance of electrochemotherapy as palliative therapy for patients with head and neck cancer and malignant melanoma and its relation to early skin reaction

**DOI:** 10.1016/j.bjorl.2023.101365

**Published:** 2023-11-22

**Authors:** Miguel Caballero-Borrego, Sandra Coll, Pilar Navarrete

**Affiliations:** a Hospital Clínic Barcelona, Department of Otorhinolaryngology, Universitat de Barcelona, Barcelona, Spain; b Universitat de Barcelona, Department of Medicine, Barcelona, Spain

**Keywords:** Electrochemotherapy, Head and neck cancer, Malignant melanoma, Palliative treatment, Bleomycin, Healing response

## Abstract

**Objectives:**

To evaluate the efficacy and tolerance after the electrochemotherapy treatment for local therapy of cutaneous and subcutaneous metastases of head-and-neck tumors and malignant melanoma refractory to standard therapies, mainly in neck metastasis of squamous cell carcinoma. And, to evaluate the relation of this response according to the skin reaction (healing with ulcer or dry crust).

**Methods:**

prospective pase II, observational clinical study of 56 patients with metastases of head-and-neck squamous cell carcinoma (*n* = 13), papillary thyroid carcinoma (*n* = 4), adenoid cystic carcinoma of parotid gland (*n* = 1) or malignant melanoma (*n* = 37, 5 in head). Patients were treated by electrochemotherapy (application of electrical pulses into the tumor) after the administration of a single intravenous dose of bleomycin. Kaplan-Meier curves were performed. The statistical significance was evaluated using log-rank test; *p*-value of less than 0.05 was considered as significant.

**Results:**

Overall clinical response was observed in 47 patients (84%). Local side effects were mild in all the patients. Ten patients (76.9%) with neck metastasis of squamous cell carcinoma had some degree of response, but only in one was complete. Patients even with only partial response had a higher overall survival than patients without response (*p* =  0.02). Most of the patients with squamous cell carcinoma had diminution of pain and anxiety. Response rate and overall survival was higher in MM patients (86.5%) than in squamous cell cancer patients (76.9%) (*p* =  0.043). The healing process (dry crust/ulcer) was not associated with the overall survival (*p* =  0.86).

**Conclusions:**

Electrochemotherapy is associated a higher overall survival and diminution of pain and anxiety. Therefore, it is an option as palliative treatment for patients with neck metastasis of squamous cell carcinoma refractory to other therapies or even as a concomitant treatment with newer immunotherapies. The type of healing of the surgical wound could not be associated with a higher rate of response or survival.

**Level of evidence:**

III.

## Highlights

• Electrochemotherapy treatment is a well tolerate procedure.
• Electrochemotherapy treatment is associated with a higher overall survival in patients with neck metastasis of squamous cell carcinoma.
• Electrochemotherapy treatment is associated with a diminution of pain and anxiety in patients with neck metastasis.
• Electrochemotherapy is an option as palliative treatment for patients with neck metastasis of carcinoma refractory to other therapies.
• The type of healing (ulcer vs dry crust) of the surgical wound could not be associated with a higher rate of response or survival.

## Introduction

With advances in the treatment of metastatic cancer, such as immunotherapy, patients live a long time and can develop the complications of advanced disease, such as Progression or Cutaneous or Subcutaneous Metastases (CSPMs). CSPMs can cause considerable morbidity and have a negative effect on quality of life. In addition, immunotherapy is ineffective or contraindicated in approximately 15% of patients, such as patients with autoimmune diseases or patients with chronic viral hepatitis, for example.^1^

For patients with advanced cancer and CSPMs lesions not controlled by standard treatments, one of the therapeutic options is the application of local-directed therapy, such as photodynamic therapy, radiotherapy, intralesional therapy, topical therapy and Electrochemotherapy (ECT), to yield improved local disease response and symptoms palliation.^2^, ^3^, ^4^ A meta-analysis on 47 prospective studies of treatment of cutaneous metastases showed the same, or even superior, effectiveness of electrochemotherapy over other therapies, such radiation, photodynamic, intralesional, and topical therapies. Even, there are authors that defense the combination of immunotherapy and ECT for squamous cell carcinoma of the head and neck,^5^ especially in bulky tumors,^6^ and for melanoma.^7^

ECT is a relatively new local ablative technique that consists in electroporation of cells in tumors through the administration of short and intense electric pulses in the tumor through special electrodes to increase the permeability of cells membranes and optimize the absorption of poorly permeable and highly intrinsic hydrophilic cytotoxic drugs (bleomycin or cisplatin).^8^ Other mechanisms of ECT are prolongation of drug entrapment in tumors due to a transient induced reduction of tumor blood flow, and a vascular disruption effect. ECT has also shown a very low toxicity profile and high patient acceptance.^4^, ^9^, ^10^, ^11^

In patients with local cutaneous or subcutaneous progression or local metastases with no further standard treatments, ECT may be an option, especially in those cases with bulky tumors that cause pain and anxiety in patients. ECT has been described by our group as an effective treatment of CSPMs of various types of solid cancer regardless the histological origin, such as squamous Head and Neck Cancer (HNC),^12^ Papillary Thyroid Carcinoma (PCT),^4^ and Malignant Melanoma (MM).^11^, ^13^

**Figure 1:**
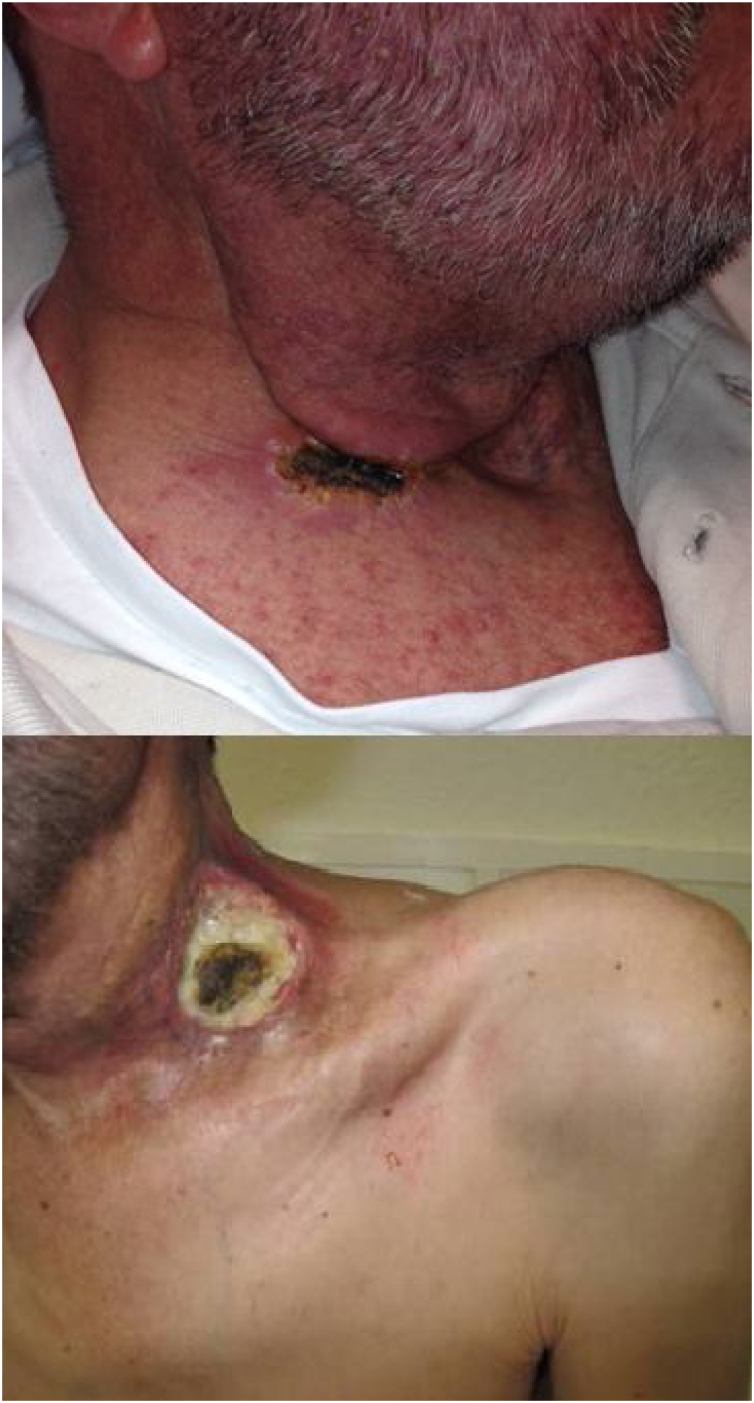
Electrochemotherapy early skin reaction: Dry crust (up) or Ulcer (down).

The immune response plays an important role during acute wound healing.^14^ After applying the ECT, our group have observed two types of the early skin reaction, a dry crust or an ulcer (Fig. 1). Ulcers may be associated with slow healing, increased pain and may be a source of infection, which could means a worse prognosis.^4^, ^14^

The first objective of this study is to evaluate the efficacy and the tolerance of ECT for palliative locally advanced head-and-neck cancer refractory to other local and systemic therapies, focusing mainly on squamous cell neck cancer metastases. And as second objective, to evaluate the efficacy and tolerance in relation to the early skin reaction (dry crust or ulcer), to consider it as a surrogate marker of the immune system status that can be used as a prognostic factor.

## Methods

### Patients

The design of this study was a prospective phase II, observational clinical study of administration of bleomycin combined with electroporation in patients with recurrent and/or metastatic head and neck cancer (HNCs, thyroid papillary carcinoma or MM in stage IV presenting one or more CPSMs as well as locoregional recurrence, or with progression of lesions and with a minimum follow-up of 5 years. Our institution participates in the European Research on Electrochemotherapy in Cancer (EURECA) project and work as part of the International Network for Sharing Practice in Electrochemotherapy (INSPECT) Network database (ISRCTN registry nº 30427). Patients had to have no other standard treatment options left, severe comorbidities or declined offered treatments. Inclusion and exclusion criteria are shown in Table 1 following EURECA guidelines for mucosal and skin cancers.^12^, ^13^

**Table 1:** Inclusion and exclusion criteria for the application of electrochemotherapy.

Inclusion criteria	Exclusion criteria
1. Histologically verified cancer of any type	1. Lesions not suitable for electrochemotherapy (bone invasion, carotid artery infiltration, etc.)
2. Progressive and/or metastatic disease	2. Active respiratory disease or serious chronic pulmonary disease
3. Primary disease not eligible for surgery for patient’s general conditions or for the need of extensive surgery	3. Severe coagulation disorders not correctable
4. Measurable lesions suitable for application of electric pulses	4. Previous allergic reactions to bleomycin
5. Age > 18-years	5. If cumulative dose of 240,000 IU BLM/m^2^ was previously reached
6. Performance status (Karnofsky ≥ 70; WHO PS ≤ 2)	6. Previous allergic reactions to bleomycin
7. Life expectancy > 3-months	7. Chronic renal dysfunction (creatinine > 150 mmoL/L)
8. Absolute white blood count above 4000 cells/L, hemoglobin greater than 10 g/dL, platelet count above 100,000 L	8. Pregnancy or lactation

Primary endpoints were local control of the treated lesion, safety and toxicity, quality-of-life, and progression-free and overall survival. Secondary endpoint was to evaluate the influence of the early skin reaction in progression-free and overall survival.

All included patients were presented at a multidisciplinary board with participation of head and neck surgeons or dermatologists, radiation oncologists, and medical oncologists where treatment plan was discussed and decided together.

### ECT treatment protocol

The patients were treated by ECT using bleomycin intravenously in a single dose followed by the application of electrical pulses into the tumor by an electrode (model N-30-HG; IGEA SRL, Carpi, Italy), powered by a commercial pulse generator for electroporation treatments. Techniques were undergone by an otorhinolaryngologist, or a dermatologist and evaluation was performed by a medical oncologist for a period of at least 5 years.

Under total anesthesia, with an inspired Oxygen Fraction (FiO_2_) of 36% or less, bleomycin was given intravenously (15–20 mg/m^2^) not exceeding 30 mg as total dose. Electric pulses were applied to the tumor nodules 8 min after the intravenous injection of bleomycin. Electric pulses were applied homogenously by hexagonal needle electrode to all the volume of superficial tumor nodules and to deeply tumors (subcutaneous nodules of maximum depth 3 cm). Electrical parameters were the same for each type of tumor at least 10 electric pulses of 100 μs with 1000 V/cm of amplitude, delivered at 1 Hz repetition frequency.

This study was approved by the Institutional Review Board (Hospital Clínic August Pi i Sunyer Biomedical Research Institute (IDIBAPS)-EUDRACT: 2013-002647-29). All patients signed the informed consent form.

### Collection of clinical data and response criteria

All patient data and parameters of the treatment procedure were collected in a Case Report Form (CRF) specifically developed for the study.

During the follow-up period, patients were evaluated by a physician two weeks after the surgery and at least every 2 months. At baseline, 6 and 12 weeks after ECT, the physician evaluate toxicity, adverse events, and tumor response of treatment by computed tomography scanning or MRI. Patients with non-melanoma head and neck tumors (the most voluminous tumors) were asked whether their pain and well-being/anxiety before and one month after surgery had improved, stayed the same, or worsened.

Response to treatment was classified as Progressive Disease (PD), Partial (PR) or Complete Response (CR). CR was defined as the disappearance of the tumor; PR as a stabilization of the disease or the reduction of the addition of the two maximum diameters of the tumor; and finally, PD was defined as an increase of the addition that those diameters. Local response was evaluated by the skin reaction after two weeks of the ECT session, depending on the formation of an ulcer or a dry scar. The Fig. 1 show the two types of local response.

### Statistical analysis

Statistical evaluation was carried out using the SPSS statistical software package for Windows (version 25.0; SPSS Inc., Chicago, IL, USA). *p*-value was calculated based on Chi-Square test and Fischer’s exact test for categorical variables to analyze patient’s characteristics and the Mann-Whitney test were used, as appropriate, to compare continuous variables. Kaplan-Meier curves were performed according to the primary tumor, to the type of response, to the concomitant systemic therapy (yes or non) and to remaining skin reaction. The statistical significance was evaluated using log-rank test; *p*-value of less than 0.05 was considered as significant.

## Results

### Patients’ characteristics

Fifty-six patients underwent an electrochemotherapy procedure at our institution. The pathology analysis of the tumors was: 4 papillary carcinoma of thyroid, one adenoid cystic carcinoma of the parotid gland, 37 of MM (6 in head), one cutaneous Squamous Cell Carcinoma (SCC), and 13 neck CPSMs of SCC. The primary tumor of this 13 neck CPSMs were 8 from oral cavity, 4 from hypopharynx and 1 from larynx. Patient demography and oncological characteristics are listed in Table 2. The median age was 67 years old. Patients with MM were older than patients with HNC (*p* = 0.024).

**Table 2:** Demographic of the study population and treatment results.

Variable (n)	Total (*n* = 56)	Head and neck SCC (*n* = 13)
Gender		
Male	29 (51.8)	9 (69.2)
Female	27 (48.2)	4 (30.8)

Age*	67.1 (54–78)	63.2 (45–82)

ECT sessions	104	30
1	23 (41.1)	11 (84.6)
2	21 (37.5)	2 (15.4)
>2	12 (21.4)	0 (0.0)

ECT local response		
No Response	9 (16.1)	3 (23.1)
Partial response	37 (66.1)	9 (69.2)
Complete response	10 (17.9)	1 (7.7)

Skin reaction		
Ulcer	25 (44.6)	9 (69.2)
Crust	31 (55.4)	4 (30.8)

One hundred and four cycles of ECT were given to the 56 patients. All the patients received at least one session of ECT, whereas 21 patients (37.5%) underwent 2 cycles and 12 (21.4%) received more than 2 sessions. Most patients with CPSM of SCC underwent a single session, but in two cases they received a second session, one for achieving a complete response and one for a minimal response and toxicity after the first session.

### Control of local disease of the tumors treated with ECT

Clinical Response (CR or PR) was observed in 47 out of 56 patients (84%) within the first three weeks after ECT (Table 2). Ten patients (76.9%) with SCC-HNC had some type of response but only one with CR. Response rate was higher in MM patients (86.5%) than in SCC-HNC patients (76.9%) (*p* =  0.043).

### Healing process, pain, and anxiety

Local side effects, such as local pain, oedema, and erythema of the subjacent skin, were mild in all the patients. A great majority of symptoms (86.5%) were resolved in the first 48 h after the procedure. Only 7 patients (13.5%) developed systemic side effects, consisted of nausea and vomiting. One of the patients of SCC group suffered from toxicoderma due to anesthesia and another patient, with chronic obstructive pulmonary disease, died the first week after de ECT due to respiratory failure unrelated to bleomycin. Of the 18 cases of non-melanoma head and neck tumors, 11 (61.1%) expressed decreased pain, 6 (33.3%) felt no change, and one (5.6%) worsened. Of these patients, 14 (77.8%) expressed an improvement in their anxiety, 3 (16.7%) reported no change, and 1 (5.6%) was worse.

Between one and three weeks after ECT sessions, the targeted lesions showed a dry crust in 31 patients (55.4%) and an ulcer in the 25 other patients (44.6%) in the skin contiguous to the tumor (*p* =  0.046). In SCC-HNC group, 9 patients (69.2%) suffered an ulcer and 4 (30.8%) a dry crust. No relation was observed between the healing process and response (*p* =  0.20).

### Survival

Table 3 shows the main overall survival results. No differences were observed according to gender (*p* =  0.84), but patients younger than 65 years showed a lower survival (*p* =  0.03). All patients were ECOG permanence status scale Grade 1 except for one patient with Grade 2. This patient had a non-statistically significant lower survival than the mean of the grade 1 patients (*p* =  0.70).

**Table 3:** Overall survivals.

Variable (*n*)	Survival (months)	95% Confidence Interval (months)	*p*
Gender			0.84
Male	14.07	3.77‒6.23	
Female	14.19	3.81‒8.19	

Age			0.03
≤65 years	10.77	4.15‒5.84	
>65 years	19.29	3.96‒16.43	

Performance status^a^			0.70
1	14.65	3.96‒16.43	
2	5.00		

Histology			0.03
Non-melanoma	12.263	4.96‒19.56	
Melanoma	25.129	17.70‒32.56	

ECT local response			
No response	6.11	1.34‒10.88	< 0.01
Partial response	19.44	13.29‒25.59	
Complete response	36.80	20.86‒52.73	

Skin reaction			0.87
Ulcer	15.17	7.53‒22.81	
Crust	13.18	6.62‒19.74	

a ECOG Performance Status Scale.

**Figure 2:**
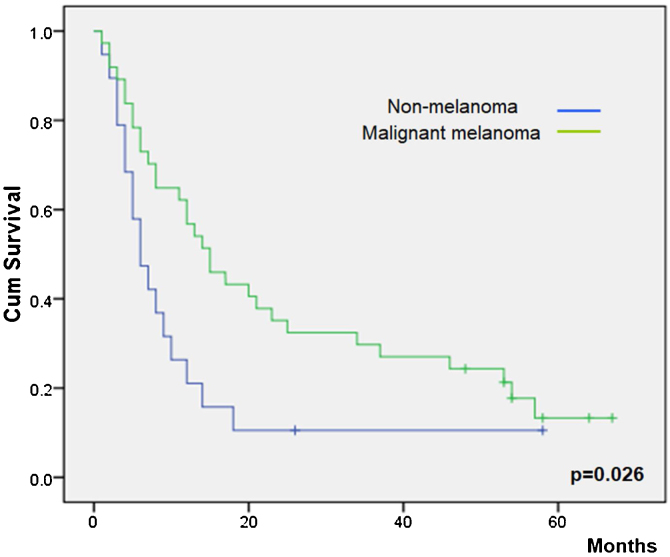
Overall survival rate according to type of primary tumor.

**Figure 3:**
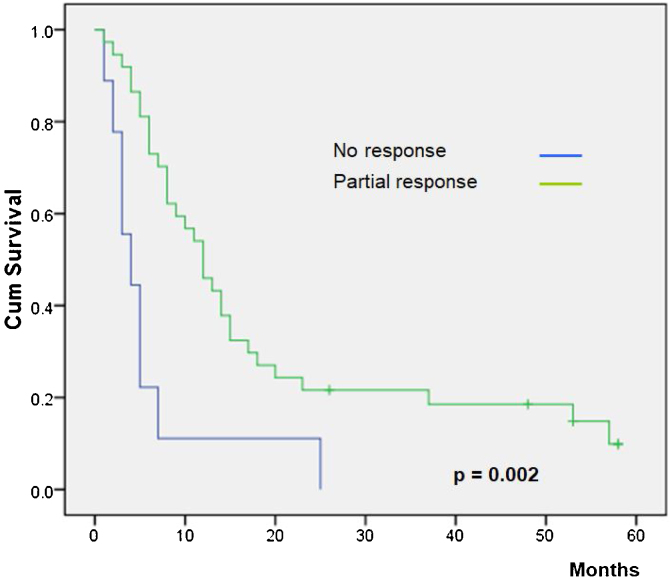
Overall survival rate according to electrochemotherapy response.

Overall survival was higher in MM patients than in non-MM patients (*p* =  0.03, Fig. 2). Obviously, patients with complete response have a higher overall survival in both the whole group and the SCC group (*p* <  0.01), but this longer survival was also observed in patients with only partial response versus no-responding patients in the whole group (*p *< 0.01, Fig. 3) and the SCC group (*p* = 0.01). We could not demonstrate a different overall survival according to the type of healing (*p* =  0.87).

## Discussion

ECT provides local control of cancers not suitable for surgery and resistant to chemo/radiotherapy.^5^ The publication of the ESOPE facilitated a broad acceptance of ECT for the treatment of CSPMs of various types of solid cancer regardless the histological origin.^8^ ECT sessions could be repeated several times to maintain local control of the disease due to immediate clinical response, low toxicity of the treatment, and lack of major side effects.^5^ Recently, the immunotherapy has gain importance in the treatment of palliative patients, but there is cases on it is contraindicated or ineffective and ECT continues been a good option.^1^ Moreover, in some cases both therapies can be applied together.^5^, ^6^, ^7^

ECT was well tolerated by our patients and majority was satisfied with the palliative results. Several authors reported up 90% of patients would accept retreatment if necessary.^15^ In our study we confirmed a lack of severe local side effects during the patients’ follow-up. In our opinion, the perceived pain and anxiety are related to the volume of the tumors, especially in the head and neck, and they improve when the volume of the tumors decreases after ECT treatment. Recently, like our study, other ECT studies have demonstrated that ECT ensures a good pain and bleeding control in skin and mucosal head and neck tumors without worsening of quality-of-life tests.^16^

In addition, the cost-benefit ratio is favorable because bleomycin is an economic drug and the technology necessary for ECT is less expensive than any ionizing radiation technology. Therefore, ECT is an ideal therapy in time of budget constraints by all health systems, and specifically for small hospitals or developing countries unable to afford the costs of other more onerous therapies.^17^

Although ECT seems to be a good option for the treatment of palliative tumors, it has little impact on poor prognosis of these patients due to the advanced cancer of these patients. In our study we analyzed a cohort of patients with CSPMs of SCC in neck refractory to other local and systemic therapies. We observed best responses rates, even with one complete response. The results are worse than those patients with MM, because probably the more superficial lesions in patients with MM than HNCS patients permit a better treatment with ECT. However, in our cohort with SCC tumors, ECT demonstrated his utility because even these patients with solo partial response showed a higher survival that patients without response. Other authors have described also benefits and complete responses in mucosal head and neck tumors.^1^, ^12^

Since the beginning of ECT some meta-analysis about this therapy have been reported.^2^, ^18^, ^19^ Whereas one of them reviewed other local therapies in addition to ECT,^2^ all of them concluded that ECT is an effective and safety treatment for CSPMs regardless the histological origin.

There are several studies available about ECT efficacy in patients with CSPMs of HNC. In HNC experiences, the response rates are lower, to 56%–85%.^12^, ^20^ In our study we showed 78.9% of clinical response the three weeks after ECT, close to the highest published rates. Our good results can be explained because we considered as a partial response those lesions with a central necrosis after the ECT procedure with stabilization of the tumor limits (stabilization of the lesion). This is not exactly how was reported in RECIST criteria that only measured de maximum diameter of the lesion to classify the clinical response. This is a problem observed by other authors and that promote another type of classification for the clinical response.^11^

In clinical observance we observed two types of healing, patients who developed either a dry crust or an ulcer. One of our hypotheses was that the type of healing could be related with the outcome. It is well known that the immune response plays an important role during acute wound healing then the local response could be related with the fitness of the immune system.^9^, ^14^ The activation of immune cells and factors initiate the inflammatory process, and promote subsequent tissue healing involving the formation of granulation tissue, neovascularization and re-epithelialization.^21^, ^22^ Finally, acute wound healing concludes with the remodeling phase, during which the granulation tissue is substituted with a scar/crust.^23^ However, dysregulation of the immune system during the wound healing process leads to persistent inflammation and delayed healing,^24^ which ultimately result in ulcer wounds.^14^, ^25^ Patients with a dry scar/crust had a lower rate of local side effects, such as local infection, comparing with the other patients. Therefore, it could be hypothesized that the dry scar/crust might be a predictor factor for local and systemic response and might be correlated with a better clinical evolution than patients with an ulcer. In spite that, in our study, those patients with a dry crust had better tolerance of treatment, and higher sensation of response (including the unique case with complete response) we cannot demonstrate a higher survival than patients with ulcers. Studies with a higher number of patients must be necessary.

Our study has also several limitations. A control group (for example patients with SCC-HNC treated with other local therapies) was not included due to other standard local therapies do not exist. Moreover, the small sample of patients with HNC precludes obtaining definitive conclusions.

## Conclusions

ECT is associated a higher overall survival, therefore is an option as palliative treatment for patients with neck metastasis of squamous cell carcinoma refractory to the other available therapies or even as a concomitant treatment with newer immunotherapies. It is a safe method with a very low toxicity due to the lower doses of bleomycin and the lack of severe side effects. Therefore, it can be used many times in the same patient with a good safety profile. ECT is well tolerated and proportionate a diminution of pain and anxiety of the patients.

The type of healing of the surgical wound could not be associated with a higher rate of response or survival.

## Funding

This study was supported by a research project grant from the Spanish Ministry for Science and Innovation (PI09/90664) and by a research project grant from the Spanish Ministry of Health, Department of Pharmacy and Health Products (EC10-067).

## Conflicts of interest

The authors declare no conflicts of interest.
